# An agent-based approach for the application of nature's forms to product conceptual design

**DOI:** 10.1371/journal.pone.0208930

**Published:** 2018-12-11

**Authors:** Dolores Parras-Burgos, Daniel G. Fernández-Pacheco, Francisco Cavas-Martínez, José Nieto Martínez, Francisco J. F. Cañavate

**Affiliations:** Department of Graphical Expression, Technical University of Cartagena, Cartagena, Spain; Zapadoceska univerzita, CZECH REPUBLIC

## Abstract

Given current high market competitiveness, it is necessary to differentiate between products that perform the same function. For this objective, designer can recur to various sources of inspiration in the searching of the more attractive form during the conceptual design stage. One of these sources can be nature, which offers a large number of geometries and textures that can be used from a shape point of view to help the designer in the creative process. This work presents an agent-based approach for a design-aided tool to provide users with some ideas, beginning with simple parts/concepts, and then increasing the complexity level according to the answers offered by designer. The proposed paradigm was implemented using the JADE agent-based platform. In order to validate the platform, several product categories were offered to fifteen different users and a total of sixty design proposals were obtained with the aid of the platform. After evaluating all the proposals, twelve of the sixty designs were finally selected and modelled by a Computer-Aided Design software. The obtained results demonstrate the feasibility of using an agent-based approach to obtain an adaptive intelligent solution to the product conceptual design problem.

## Introduction

### Creative and bioinspired design process

It is difficult to know when a product will be successful in the market because it is necessary to take into account the relationship that links objects, human beings and the environment, and to highlight the different product functions to achieve this. Three groups of a product’s characteristic functions are differentiated: shape-aesthetic, symbolic-communicative and practical [1, 2]. Therefore, design is a creative process in which the components of functionality, aesthetics and usability of the product must be taken into account [3]. However, creative activity is not infinite and abstract, but is conditioned by designers’ characteristics: spatial vision and imagination; curiosity, observation and investigative mood; discipline and creativity; technical and scientific training [4].

A designer’s aim is to create shapes, materials, textures, colours and structures that improve our surroundings by producing utilitarian objects, machines and tools that satisfy our needs and comforts [4]. Industrial design can be defined as a creative act related to the design of objects as it reconciles the functional aspect with the aesthetic function. Creativity, in turn, allows us to generate new ideas or concepts, and to associate already known ideas and concepts that can usually produce original solutions [5]. When an industrial designer faces the challenge of finding a shape-based solution to design a product, resources or sources of inspiration may be needed to help find the most suitable form; one of these sources can be nature. If one observes nature in a general way, it may seem to contain an infinite number of changing forms depending on the circumstances. If, however, nature is observed in more detail, a certain number of shapes and patterns are repeated by taking into account a series of properties or physical limitations. Some designers have started using inspiration based on nature for product design, who use this resource from a shape point of view to model their designs in furniture, interior design, architecture, etc. [6, 7].

In light of this, we can deduce that a product might be successful not only if it possesses aesthetic quality and intelligent functionality, but also if it emotionally satisfies consumers. Biomimetic designs are widely used in product design to emphasise an emotional interaction. Therefore, understanding the psychological effects of biomimetic products is becoming an important aspect when it comes to developing products with strong affective qualities. Some studies have analysed these types of elements, whose results indicate that consumers have different degrees of emotional responses to products with distinct levels of biomimetics [8]. In a statistical study, aesthetic consumer preferences have been analysed in relation to a product designed with golden shapes and present in nature by comparing it with others in the market. The results did not indicate relevant differences between the age and gender factors of the participants, but proved remarkable in level of education terms, where the participants with a higher level of education rated this design type with a higher score [9]. Indeed the "aesthetic intelligence" concept arises, which tells us that we possess it innately and, sometimes, unconsciously, defined as the ability to perceive a wide range of product qualities that outline our response to them. It is a way to design for senses as a means to provide products so that customers can feel a higher degree of empathy [10].

#### What can be done to enhance creativity?

Research in cognitive psychology and design thinking has shown that the generation of internal representations in images and external representations through sketches is fundamental for solving design problems. An empirical study revealed that the presence of different types of visual stimuli can affect the achieved performance, measured in terms of practicality, originality and creativity score, with designs developed by subjects and done under different conditions [11]. Cognitive studies have been conducted to evaluate the impact of biological examples during the process of generating ideas in conceptual design. The results suggest that exposure to biological examples can increase the generation of novel design ideas, as opposed to exposure with human engineering examples [5]. Cognitive science, strongly dominated by a linguistic paradigm, has yet to recognise the primordial role of visual reasoning to solve problems in many cases. The creation of design and computational tools, among others, should be optimised so as not to overlook intuitive visualisation [12].

To advance designers’ work, it is necessary to use support tools in the conceptual phase if the source of inspiration is nature. Some tools can be used to develop different types of resources with which designers and engineers can easily access some biological information that can help solve their design problems. The Chakrabarti System, with the Idea-Inspire tool [13–15] is a method that seeks to provide analogical ideas for design that can be biologically or artificially inspired. BioTRIZ allows designers without extensive knowledge about biological systems to apply it to engineering design from matrices [16, 17]. With simple cards [18, 19] industrial designers can be helped to extend their range of solutions to solve problems through biomimicry. There are also dictionaries [20, 21] that relate engineering and biology terms, and CAD systems that provide access to functional models of biological systems [22]. One of the best-known tools is Ask Nature [23–25], which is a database of "biological patents" in which a wide range of biological mechanisms and properties can be searched to solve technological problems.

Table 1 shows a comparative table of several tools that, from a functional point of view, proposes solutions inspired by nature to solve specific problems that may arise. However, no previous tool has analysed shape and aesthetic aspects, which makes it impossible to locate those elements that can improve the creative process in a bioinspired way. In the specific case of the biomimetic cards [18, 19], although solutions are offered from a shape point of view, they are actually used to solve functional aspects within specific categories. Therefore, a methodology is necessary to provide users with a series of initial ideas that will shape and increase the complexity level according to the decisions made by designers. Given the many possibilities and the complexity of the proposed resolution, a valid solution would be to resort to using agent-based systems.

**Table 1:** Comparison of some current toolys to support bio-inspired design.

Authors Reference	Name	Functional aspects	Shape aspects	Aesthetics aspects	Description
**[24], [25]**	Ask Nature (Web—Online Database)	YES	NO	NO	2406 functional characteristics obtained from elements of nature.
**[16], [17]**	Triz / BioTriz (Database)	YES	NO	NO	Through a matrix, solutions are related to similar principles.
**[18], [19]**	Biomimetic cards	YES	YES	NO	Four categories of biomimetics are defined in relation to design: materials, mechanical / dynamic, structures and shapes.
**[13], [14], [15]**	Idea-Inspire (Database)	YES	NO	NO	Database with more than 700 entries of natural and artificial phenomena that can be searched using 7 attributes.
**[22]**	DANE (Design by analogy to nature engine, CAD System)	YES	NO	NO	DANE is a CAD system that provides access to functional models of biological systems. It takes into account four characteristics: cognitive, collaborative, conceptual and creative.
**[20]**	Method	YES	NO	NO	Method to unite systematically the disparate domains of biology and engineering using natural language analysis.
**[47]**	The BioM Innovation Database	YES	NO	NO	The global biomimetic activity is shown since 1960. It is the first tool to include data related to the process, focus, geography and vocabulary.
**[21]**	The engineering-to-biology Thesaurus	NO	NO	NO	Dictionary that relates terms of engineering and biology as an aid in the teaching of biomimicry in engineering.
**[48], [49]**	Method	YES	NO	NO	Use of descriptions of biological phenomena to develop concepts to solve a simple problem.

### Multi-agent systems

Multi-agent systems have proven to be really powerful tools for modelling and understanding phenomena in several fields, such as economics and trading, health care, urban planning and social sciences. They are integrated by multiple interacting intelligent agents with some degree of autonomy, and are able to cooperate, compete, communicate and act flexibly to fulfil defined objectives [26].

Their use is increasingly extending, and some works about product design can be found in the scientific literature; e.g., the agent-based recommender system developed by Moon et al [27] to develop customised families of products; the agent framework presented by Pinho et al [28] to improve process awareness in an architecture company. Li et al [29] also resort to multi-agents to create a risk assessment model for complex product designs. Liu et al [30] define a multi-agent-based architecture to support collaborative product lightweight design. Madhusudan [31] presents an agent-based process coordination framework for distributed design process management. Ballouki et al [32] address the supply chain configuration problem by considering new product re-design with the help of a multi-agent system.

Nevertheless, no advances in the conceptual design stage have yet been found. Only some works based on functional aspects are available in the scientific literature, such as the agent-based framework defined by Cao et al [33] to guide the conceptual design of mechanical products, and the agent-based framework presented by Soni et al [34] for the conceptual design generation stage of the industrial product design process. However, these works do not consider nature as a source of inspiration, and no shape or aesthetic aspects are analysed and offered to designers in the initial conceptual stage.

For all these reasons, this paper presents a new agent-based approach to provide users with some ideas based on shape and aesthetics aspects in the conceptual design stage, and begins with basic symbols/concepts and increases the complexity level according to the answers received from designers. The proposed paradigm permits an intelligent and adaptive solution based on nature-inspired forms to be obtained for the product conceptual design problem.

## The paradigm

The proposed creative process is based on using analogy relationships, where different elements or concepts from several areas are compared to extract a conceptual form to be applied to design a product.

**Fig 1:**
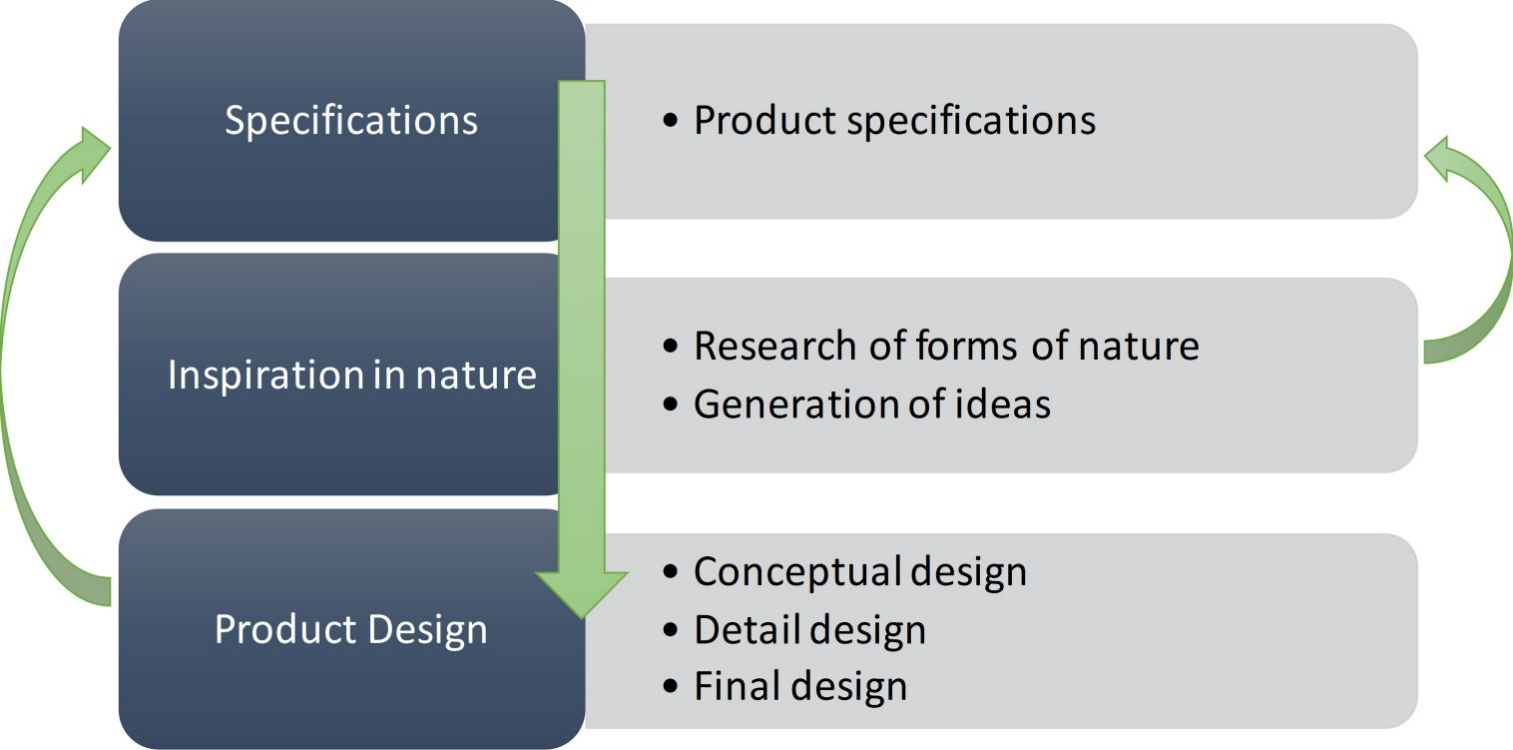
Flowchart of the design process.

In this context, a methodology based on the search for nature-inspired forms is not a unidirectional process because it may require several iterations, and does not have a single solution as different conceptual alternatives to be obtained. Finally, that which best fits the specifications is selected (Fig 1).

**Fig 2:**
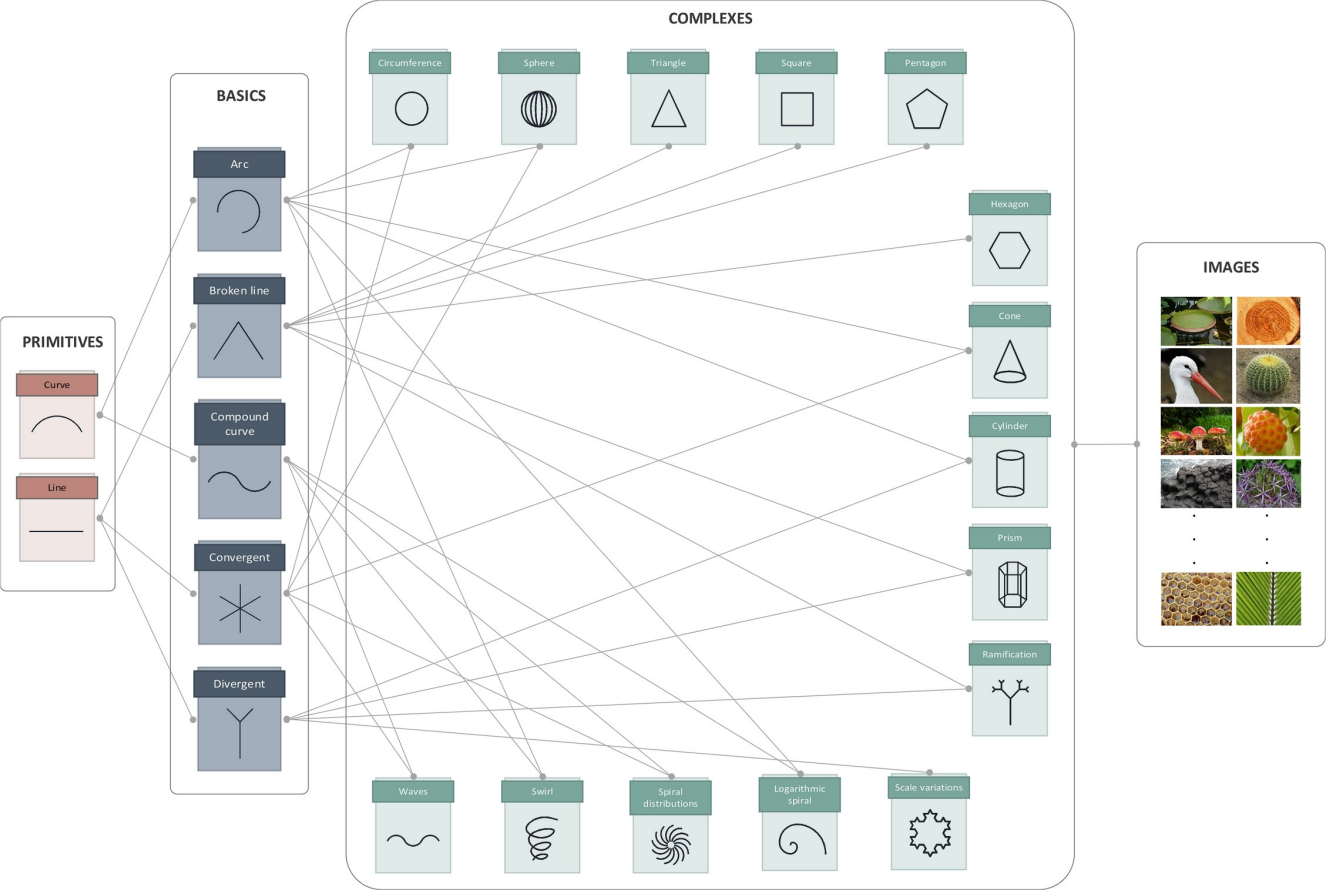
Relations between the symbols at each level.

The paradigm proposed here is based on a hierarchical decomposition of geometric symbols according to different stages or levels to help users specify the idea they are looking for during the conceptual design of the product. Thus in this first approach of the paradigm, the first level is composed of two primitive symbols, the second level contains five basic symbols, the third is formed by fifteen complex symbols and the fourth level comprises ninety images of different elements from nature. All these symbols and images are related to one another according to the scheme shown in Fig 2.

**Fig 3:**
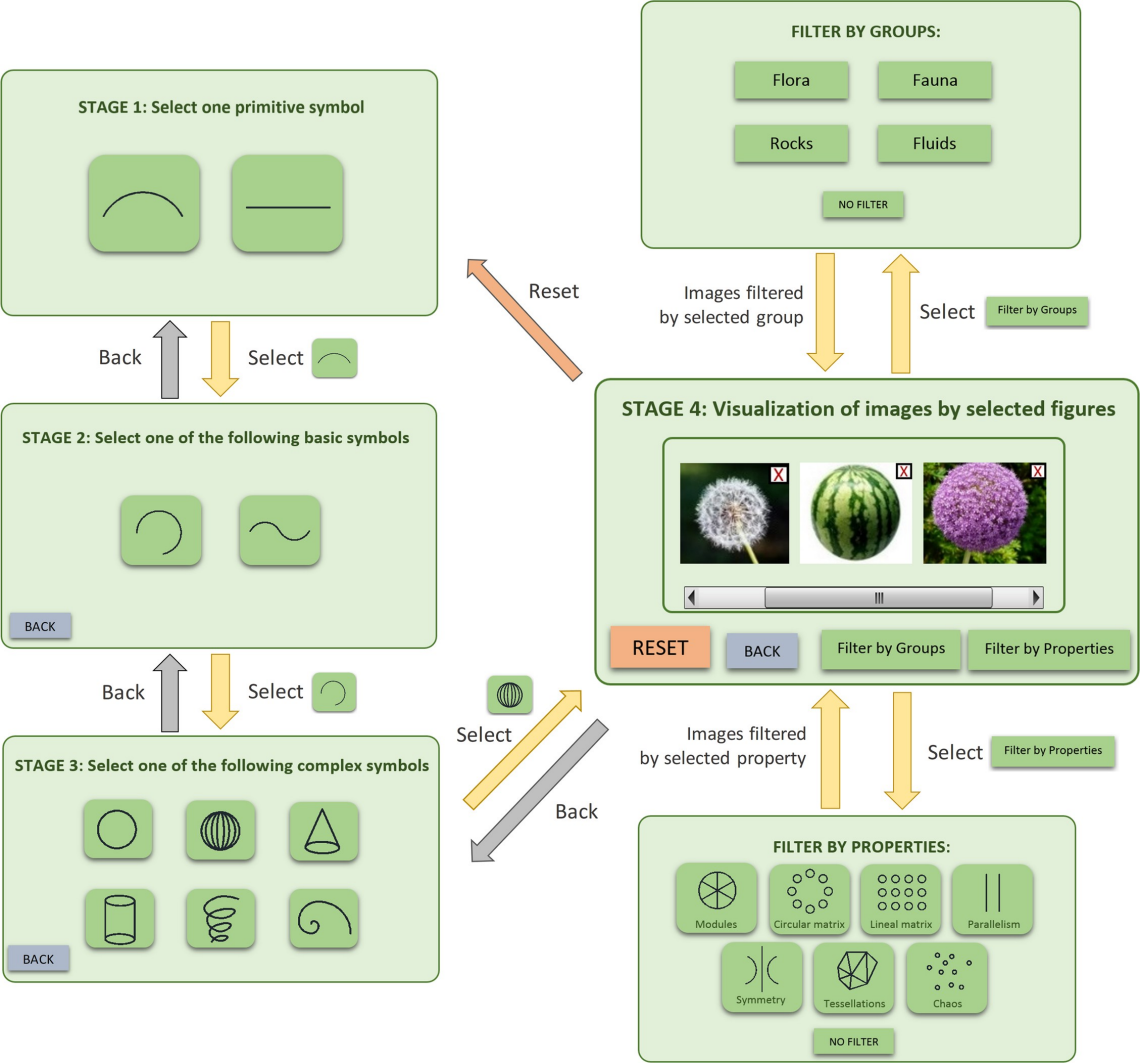
Functioning scheme of the paradigm.

The operation of this cognitive process is based on selecting those symbols that suggest ideas to help continue advancing in each stage. The selection of a symbol in a determined stage implies that only those symbols at the higher level are related to the selected symbol shown in the next stage. In this way, each choice offers designers new search alternatives. Fig 3 illustrates an example of the operation of the proposed paradigm, where we can see that the symbols shown in each stage depend on previous selections.

While stages 1, 2 and 3 show graphical symbols, which suggest specific geometric shapes, stage 4 offers a certain number of images of elements from nature whose shape and aesthetic characteristics are related to those symbols (Fig 3). Having reached this fourth stage, the possibility of eliminating those images that do not suggest any idea at that time is offered. This fourth stage also provides the possibility of applying a group filter to the results to differentiate between elements of nature (fauna, flora, rocks and fluids), and a properties filter to classify according to their distribution and position in nature (modules, circular matrix, linear matrix, parallelism, symmetry, tessellations and chaos). By applying these procedures, the number of results lowers and designers can gradually specify their creative process. Finally, if designers have not found the ideas they were looking for during the process, they have the option of starting all over again from the beginning by pressing a "reset" button (Fig 3). Likewise, if designers wish to go back to any stage because the given results do not fit their creative concept, they can press a "back" button to return to the previous stage and to make new decisions.

## The agent-based architecture

**Fig 4:**
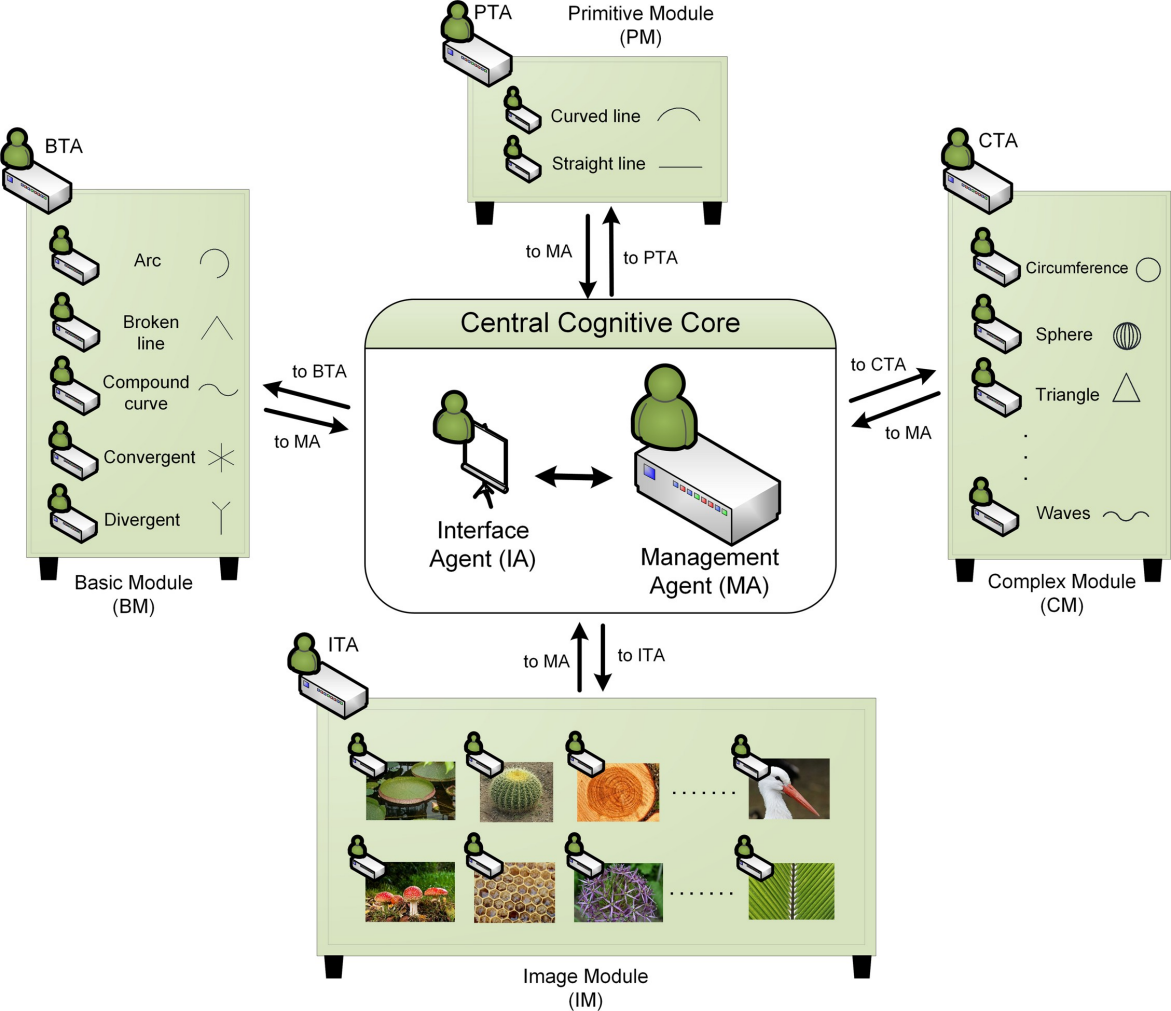
Agent-based architecture of the proposed paradigm.

The very nature of a multi-agent architecture offers scalable solutions that enable the proposed paradigm to be extended in future developments. For this reason, we chose a modular architecture (Fig 4) composed of a Central Cognitive Core and several Modules that contain the different symbols and images corresponding to each decision stage. All these modules are managed by the so-called Talking Agents, which communicate with the agents that comprise their respective modules. Hereafter, each component is described in detail, along with a complete execution cycle to provide a better understanding.

### Central cognitive core

Inside this component, two agents work jointly to permit the platform to function properly.

The Interface Agent (IA) is responsible for managing the user interface, shows designers the possible options available in each stage and captures their selections.

The Management Agent (MA) receives the selections entered by the user through the IA and communicates them to the corresponding Talking Agents of each module, as appropriate. In the same way, after receiving the information from the Talking Agents, the MA analyses it and proceeds to communicate the changes to be displayed in the user interface to the IA. Given this agent’s heavy workload, it is assigned a larger icon in Fig 4.

### Primitive module

This module is managed by the Primitive Talking Agent (PTA), which will be in charge of communicating with the Central Cognitive Core, and more specifically with the MA, and of transmitting the received information to its Primitive Agents. Each one of these Primitive Agents starts in an *Active* state at the beginning of the process. Once the user has made a selection in the first stage, the agent that corresponds to the selected primitive symbol changes its state to *Selected*, while the rest remain in the *Active* state.

### Basic module

Similarly, the basic module is managed by the Basic Talking Agent (BTA), which will be responsible of receiving information from the Central Cognitive Core about the primitive symbol selected by the user and of communicating it to its five Basic Agents. These agents, which start from an *Idle* state at the beginning of the application, change their state to *Active* or *Asleep* depending on the primitive symbol received. After all the Basic Agents have established their states, the Central Cognitive Core is informed about which Basic Agents remain in an *Active* state and, therefore, can be shown as an option in the second decision stage. The selection of a basic symbol in this stage results in its agent changing its state to *Selected*, while the state of the other Basic Agents remains.

### Complex module

The complex module is managed by the Complex Talking Agent (CTA), which will be responsible of receiving information from the Central Cognitive Core about the basic symbol selected by the user and of communicating it to its fifteen Complex Agents. As in previous cases, these agents start from an *Idle* state at the start of the application and their state changes to *Active* or *Asleep* according to the basic symbol received. Having established the states of all the Complex Agents, the Central Cognitive Core is informed about which Complex Agents remain in the *Active* state and can, therefore, be shown as an option in the third decision stage. The selection of a complex symbol in this stage leads its agent to change its state to *Selected*, while the state of the other Complex Agents remains.

### Image module

In the same way, the image module is managed by the Image Talking Agent (ITA), which will be responsible of receiving information from the Central Cognitive Core about the complex symbol selected by the user and of communicating it to its ninety Image Agents. The state of these agents, which start from an *Idle* state when the application starts, changes to *Active* or *Asleep* depending on the complex symbol received. Having established the states of all the Image Agents, the Central Cognitive Core is informed about which Image Agents remain in the *Active* state and, therefore, can be shown as an option in the fourth decision stage.

When the first results are shown in the fourth stage, designers may wish to apply a filter by group or properties. This action is notified to the ITA and, consequently, the Image Agents update their state by taking into account the new information. Then the Central Cognitive Core is advised to update the options shown in the fourth decision stage.

Similarly, if a designer removes any of the options shown in the fourth stage using the corresponding icon, the ITA is notified again to order the Image Agent corresponding to the deleted option to change its state to *Asleep*.

### Complete execution cycle

After defining the different agents that comprise the architecture (Fig 4), we proceed to describe a complete execution cycle. The sequence of execution is structured according to several stages:

- Stage zero: as soon as the application starts, the MA orders the Talking Agents of all the modules (PTA, BTA, CTA and ITA) to pass all their agents to the *Idle* state. These Talking Agents confirm with the MA that their agents are available and that execution can continue. Next, the MA requests from the PTA information about which agents integrate its Module by receiving the identification of all the Primitive Agents and transferring them to the *Active* state. Having received this information, the MA contacts the IA and indicates which primitive symbols should be shown in the first stage.
- Stage one: in this first stage, the IA shows all the primitive symbols that correspond to the received information and waits for the user to select one of these symbols. After a selection is made, the IA informs the MA about the selected primitive symbol, which forwards the information to both the PTA and BTA. The PTA changes the state of the Primitive Agent corresponding to the selected primitive symbol to *Active*. The BTA indicates to its Basic Agents which primitive symbol the user selected and all these agents change their state to *Active* or *Asleep* depending on its correlation with the chosen primitive symbol. The BTA then receives the states of all its agents and informs the MA about which ones remain *Active*. This information is forwarded to the IA to indicate which basic symbols should be shown in the second stage.
- Stage two: in this second stage the IA shows only the basic symbols that remain *Active* according to the received information, and awaits the user to select one of these symbols. Two situations may occur at this point:
  ■ The user selects a basic symbol: after the selection is made, the IA informs the MA about the selected basic symbol, which forwards the information to both the BTA and CTA. The BTA changes the state of the Basic Agent corresponding to the selected basic symbol to *Active*. The CTA indicates to its Complex Agents which basic symbol is selected by the user and all these agents change their state to *Active* or *Asleep* depending on their correlation with the chosen basic symbol. The CTA then receives the states of all its agents and informs the MA about which ones remain *Active*. This information is forwarded to the IA to indicate which complex symbols should be shown in the third stage.
  ■ The user presses the *Back* button: in this case, the IA notifies the MA of the user's desire to return to the previous stage, and this information is forwarded to both the PTA and BTA. The BTA changes the state of all its Basic Agents to *Idle*. The PTA modifies the state of its selected Primitive Agent from *Selected* to *Active*, and contacts the MA so that the IA once again shows the first decision stage.
- Stage three: in the third stage the IA shows only the complex symbols that remain *Active* according to the received information, and awaits the user to select one of these symbols. Two situations may occur at this point:
  ■ The user selects a complex symbol: once the selection is made, the IA informs the MA about the selected complex symbol, which forwards the information to both the CTA and ITA. The CTA changes the state of the Complex Agent corresponding to the selected complex symbol to *Active*. The ITA indicates to its Image Agents which complex symbol is selected by the user and all these agents change their state to *Active* or *Asleep* depending on their correlation with the chosen complex symbol. Next, the ITA receives the states of all its agents and informs the MA about which ones remain *Active*. This information is forwarded to the IA to indicate which images should be shown in the fourth stage.
  ■ The user presses the Back button: in this case, the IA informs the MA about the user's desire to return to the previous stage, and this information is forwarded to both the BTA and CTA. The CTA changes the state of all its Complex Agents to *Idle*. The BTA modifies the state of its selected Basic Agent from *Selected* to *Active*, and contacts the MA so that the IA once again shows the second decision stage.
- Stage four: in the fourth stage the IA shows only those images whose agents remain *Active* according to the received information. Several situations may occur at this point:
  ■ The user wishes to discard an image: for this purpose, the user clicks on the existing red-cross icon at the top right-hand area of the image to be deleted. After clicking, the IA removes the selected image from the screen and informs the MA about the discarded image, which forwards the information to the ITA. The ITA then orders the corresponding Image Agent to change its state to *Asleep*.
  ■ The user wishes to apply a filter by groups or properties: for this action, the user clicks on the corresponding button. Then a window appears where several filtering types are offered (a no filter option can also be selected). If a filter is selected, the IA informs the MA about the selected filter, which forwards this information to the ITA. The ITA then contacts all its Image Agents and asks them to update their states depending on two factors: i) the correlation with the complex symbol selected in the previous stage; ii) the inclusion, or not, in the active filters (one filtering can be applied at the same time per group and once again per properties). Next, the ITA receives the states of all its agents and informs the MA about those that remain *Active*. This information is forwarded to the IA to update the images shown in the fourth stage.
  ■ The user presses the Back button: in this case, the IA informs the MA about the user's desire to return to the previous stage, and this information is forwarded to both the CTA and ITA. The ITA changes the state of all its Image Agents to *Idle*. The CTA modifies the state of its selected Complex Agent from *Selected* to *Active*, and contacts the MA so that the IA once again shows the third decision stage.
  ■ The user presses the Reset button: in this case, the IA informs the MA about the user's desire to start the process from the beginning by going back to the first decision stage. Then the MA orders the BTA, CTA and ITA to pass all their agents to the *Idle* state, and the PTA to pass all their agents to the *Active* state. These Talking Agents confirm to the MA that their agents are available, and that the execution can continue starting again the first stage.

## Implementing the architecture

The above-described paradigm is implemented into an architecture based on multi-agents that permits the proposed system to be simulated and assessed. Several agent-based platforms have been analysed [35–37] by selecting the JADE agent-based platform [38], which is based on Java and is one of the most widely used platforms for research purposes [39–43]. On this platform, communication is in charge of the messages encoded in the FIPA-ACL standard [44], which is a communication language natively supported by JADE. FIPA-ACL specifies both message fields and message performatives, which consist of the communicative acts of requesting, informing, refusing or accepting a proposal, among others. Thus, JADE offers the means for sending and receiving messages in a manner that is transparent to the agent developer, and it is even possible to monitor the exchange of messages by using a built-in sniffer agent.

## Experimental work

**Fig 5:**
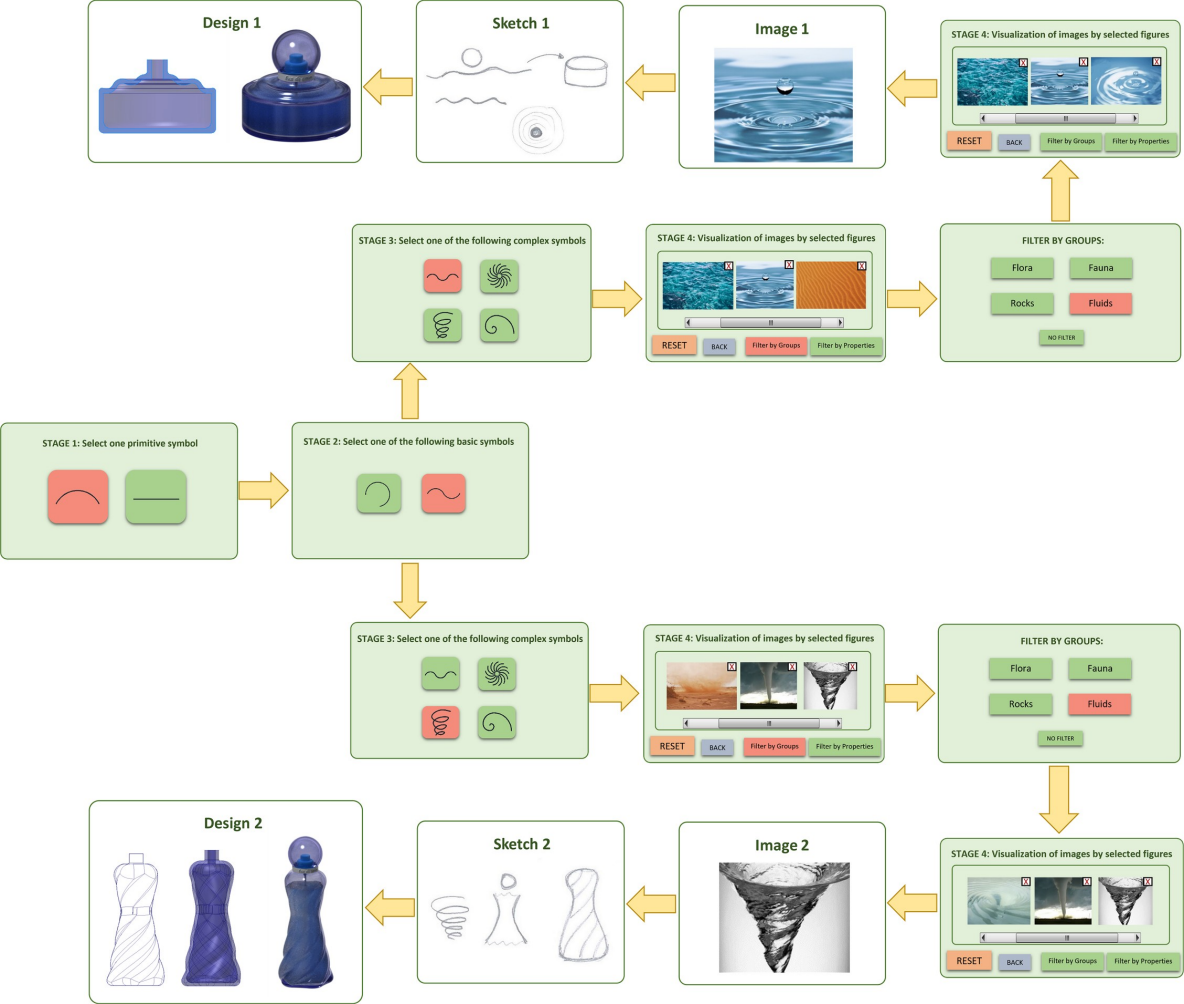
Search process for ideas to design a perfume container.

One example to help successfully capture the objective of the proposed paradigm is as follows: a user was entrusted with the task of designing a perfume container with bio-inspired forms. Fig 5 shows a summary of the choices made by the designer and the results offered by the platform. We can see that after several iterations, the images related to water in motion offer the analogy from which the shape-based concepts of perfume containers were developed. Although the initial challenge was to design a single perfume container, two ideas were finally used: a male version and a female version. The process began by selecting curved forms in stages 1 and 2, while two different symbols were analysed in stage 3: i) *waves*, which suggest the shape of the male perfume bottle with a more compact and simple appearance; ii) *water swirl*, which suggests a more stylised and feminine form for the female perfume bottle. In both designs, the idea of a stopper was unified as a drop of water without gravity (Fig 5).

**Fig 6:**
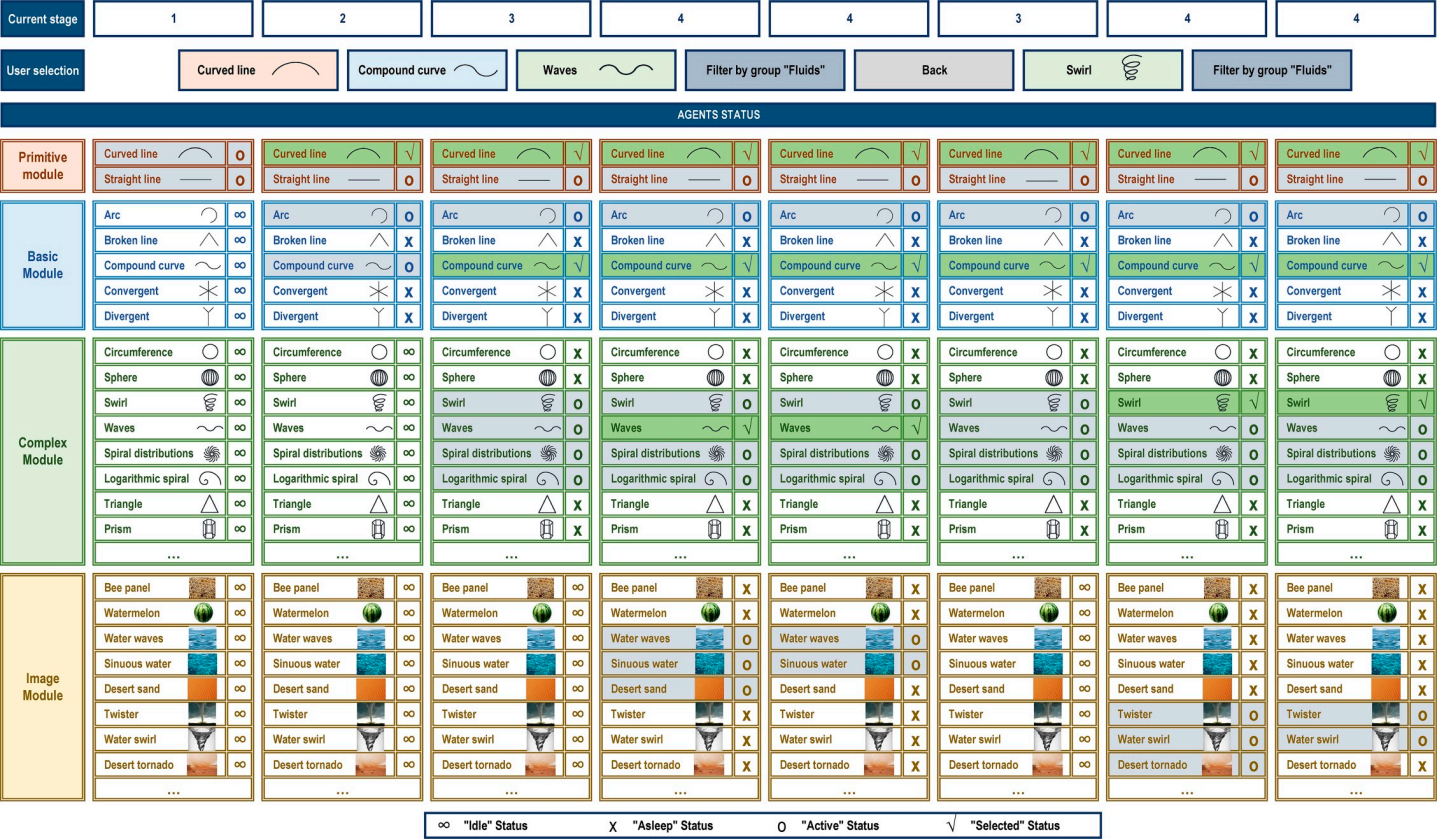
Detail of agent states when searching for ideas to design a perfume container.

In order to facilitate the understanding of the proposed paradigm for the aforementioned application example, Fig 6 provides a chronological table of the states of the agents that compose each Module. In more detail:

- Stage one: in a first stage all the Primitive Module agents are in the *Active* state as they are available to be shown in the application. However, the *Idle* state of the agents of the remaining modules remains.
  ■ Next, the user selects the primitive symbol for *curved line*, which implies: i) the Primitive Agent for *curved line* changes its state to *Selected*; ii) the Basic Agents for *arc* and *compound curve* change their state to *Active*, while the rest of the Basic Agents change their state to *Asleep*.
- Stage two: in this second stage only the basic symbols that are *Active* are shown: *arc* and *compound curve*.
  ■ The user selects the basic symbol for the compound curve, which implies: i) the Basic Agent for the compound curve changes its state to *Selected*; ii) the Complex Agents for *waves*, *swirl* and *spiral distributions* and *logarithmic spiral* change their state to *Active* and the rest of the Complex Agents change their state to *Asleep*.
- Stage three: in the third stage only the complex symbols that are *Active* are shown: *waves*, *swirl*, *spiral distributions* and *logarithmic spiral*.
  ■ The user selects the complex symbol for *waves*, which implies: i) the Complex Agent for *waves* changes its state to *Selected*; ii) the Image Agents for *waves*, *swirl*, *spiral distributions and logarithmic spiral* change their state to *Active* or *Asleep* depending on their correlation with the chosen complex symbol.
- Stage four: in this fourth stage only the images that correspond to the Image Agents that are *Active* are shown; for instance, *water waves*, *sinuous water* and *desert sand*, among others.
  ■ To filter the results, the user selects applying a filter by group by choosing the 'fluids' category. As a result, some Image Agents change their state to *Asleep*; e.g., *desert sand*.
- Stage four: the fourth stage is shown again, but this time the images that do not comply with the 'fluids' filter are no longer displayed.
  ■ At this point, the designer finds that the image for *water waves* inspires him/her to sketch a male perfume bottle. However, the user decides to go back to the previous stage to see if, by taking into account the same concept, an alternative solution can be obtained to help design a feminine version of the product. Pressing the BACK button leads to two processes: i) all the Image Agents change their state to *Idle*; ii) the previously selected complex agent for *waves* changes its state from *Selected* to *Active*.
- Stage three: when returning to the third stage, the complex symbols in the *Active* state are shown again: *waves*, *swirl*, *spiral distributions* and *logarithmic spiral*.
  ■ On this occasion the user selects the complex symbol for *swirl*, which entails: i) the Complex Agent for *swirl* changes its state to *Selected*; ii) the Image Agents change their state to *Active* or *Asleep* depending on their relation with the selected complex symbol.
- Stage four: having reached the fourth stage, only the images corresponding to the Image Agents that are *Active* are shown; for instance, *twister*, *water swirl* and *desert tornado*, among others.
  ■ To filter the results, the user selects applying a filter by group, and chooses again the 'fluids' category. As a result, some Image Agents change their state to *Asleep*, as in the case of *desert tornado*.
- Stage four: the fourth stage is shown again, but this time the images that do not comply with the 'fluids' filter are no longer displayed.
  ■ At this point, the designer finds that the image for *water swirl* inspires him/her to sketch the female perfume bottle, which ends the application.

## Results and discussion

In order to assess the paradigm, four topics (home furniture, household item, urban furniture and small household appliances) were offered to fifteen different users, and sixty design proposals that demonstrated the feasibility of the proposed agent-based platform were obtained. The participation in the study was offered as a voluntary practice/exercise for the students (ranging in age between 20 and 24 years old) of the subject Computer-Aided-Design taught in two Degrees at the Technical University of Cartagena (Degree in Mechanical Engineering and Degree in Automation and Industrial Electronic Engineering), and was performed in accordance with the ethical standards laid down in the Declaration of Helsinki (Seventh revision, October 2013, Fortaleza, Brasil). An informed consent was obtained from all subjects of the study.

The initial premise was to obtain designs of objects by prioritising the shape and aesthetics of the product instead of the functional aspect. In this way, the creative process acquires a higher degree of freedom because it is not conditioned by any prior requirement. During each test, users made different iterations by searching among all the options provided by the system until they find a given image that suggests them the shape of the object that they wish to design. After evaluating all the proposals, twelve of the sixty designs were finally selected and modelled by the Computer-Aided Design software SolidWorks v2016.

**Fig 7:**
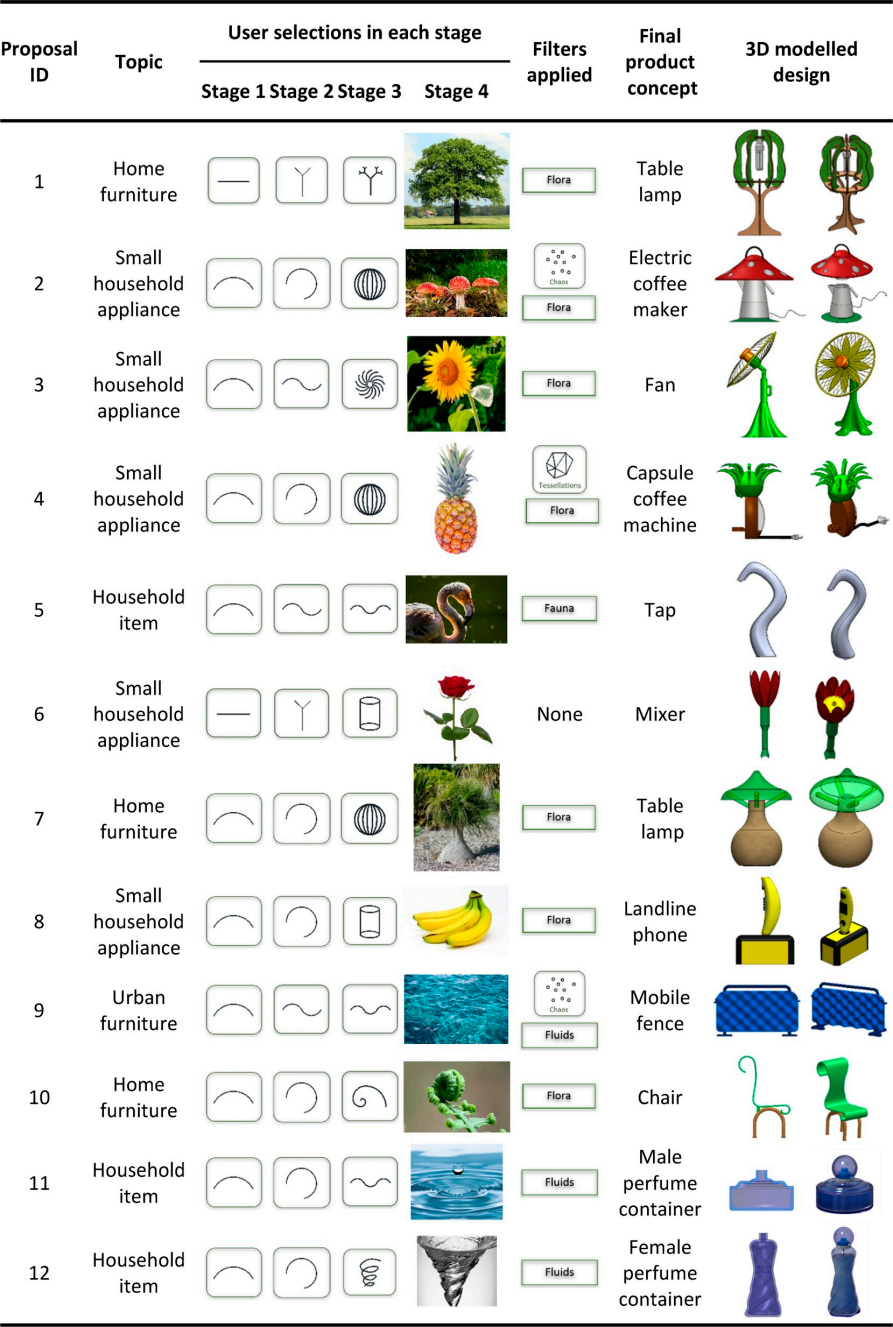
Detail of the finally selected designs during the evaluation process of the proposed paradigm.

Fig 7 provides more detailed information about the entire evaluation process: the initial topic chosen by the user, the selections made by the user in each stage, if any filter/s was/were applied, the image finally selected by the user, the final product concept and the 3D modelled design.

For proposal number one, the user chose the “home furniture” topic and started his creative process by selecting linear and branched forms in initial stages. Later he applied the filter by the “flora” group and found an image of a tree that inspired him the design concept for a table lamp. This design has the characteristic feature of being removable in flat components and can be stored without taking up much space.

For proposals numbers two, three and four, users selected the "small household appliance" topic. In all three cases, users started the creative process by selecting curved forms in initial stages. In the second proposal, the user selected the spherical form in the third stage and applied the filter by the "flora" group, and finally designed a mushroom-shaped coffee machine. In the third proposal, the user selected a spiral distribution in the third stage and also applied the filter by "flora" group to find the shape-based concept of a fan in a sunflower image. In the fourth proposal, the user selected the spherical form in the third stage and applied the filter by the "flora" group as in the second proposal but, in this case, the results were filtered by the "tessellations" property to find a very original concept for a capsule coffee machine in the image of a pineapple.

In proposal number five, the user took the "household item" topic, and selected the curved forms in the three stages, which later reduced the number of images by choosing the filter by the "fauna" group. From the proposed results, the image of a flamingo finally inspired him/her the shape for a tap.

The user of proposal number six chose the "small household appliance" topic. He started the first stage by selecting the linear element icon, and chose the shape of a cylinder in the third stage. From the resulting images, a rose with the vertical stem was finally selected to design a mixer, which provided a very different design concept from those found on the market.

In proposal number seven, the "home furniture" topic was selected by the user, who began the creative process with curved forms and chose the spherical shape for the third stage. The results were filtered by using the "flora" group and the image of a plant with an almost spherical trunk was finally selected. This image suggested the design concept of a very interesting small table lamp to be used both indoors and outdoors.

The user of proposal number eight decided to perform his creative process by selecting curved and cylindrical forms, and filtered the obtained results with the "flora" group. Finally, the image of some bananas was used for an original landline phone proposal.

In proposal number nine, the user chose the "urban furniture" topic. He finally designed a suggestive mobile protection fence by selecting curved and wavy forms in early stages, and by filtering by groups with the "fluids" option.

In proposal number ten, the selected topic was "home furniture" and the user chose curved and logarithmic forms in early stages by filtering the resulting images with the "flora" group. The final design was inspired by an image of a fern, which suggested an interesting shape for a chair.

Finally, proposals eleven and twelve were performed by the same user in the “household item” topic, who obtained two designs with different iterations. In the first stage, the user chose the curved forms for both designs. With the wave icon and by filtering by the "fluids" group, he achieved the concept of a perfume container for men. In the same line as this container, but with the swirl selection in the third stage, the chosen image suggested a container for women with a more suggestive slender shape. Both designs shared the concept of a drop of water without gravity, used for the stopper of both containers.

Once finished the evaluation phase, all users were asked about the platform and several improvement recommendations were collected, as for instance:

■ The number of images implemented for the final decision step (stage four) should be increased.
■ The implementation of a sketching tool could help the designer to save his/her ideas to be used later.
■ Include some information jointly with the geometrical symbols to be selected in every stage.
■ Every image shown in the fourth stage should include a button that, if clicked, the following data are provided:
  - Information related to the nature element itself, as well as other symbols or properties of the element.
  - 3D models predesigned and related to the geometry of the element shown, including public links to download them when possible.
  - Examples of final bioinspired product designs, what permits the designer to see a variety of applications of the selected image to real products.

In general, a satisfactory result was obtained in terms of management and everyone considered that the tool was useful and favoured the creative process in the conceptual design phase.

## Conclusions

When designers face the challenge of shaping a product, some sources of inspiration are necessary to improve the creative process. Nature can be an inexhaustible resource of ideas, but given the vast numbers of possibilities available, it can be a complex task if certain concepts are searched for. To improve designers’ work, it is necessary to use support tools in the conceptual design phase that promote creativity thanks to visual stimuli. Thus, exposure to biological examples with the help of symbols and images can enhance the generation of novel ideas. Based on this concept, a new approach is proposed to establish cognitive relations between different shape parameters of nature. The beginning of the creative process starts with symbols that represent the most outstanding forms found in nature. Once several selections have been made, the tool offers a series of images from elements of nature that meet previously established shape-based conditions. This cognitive process enhances creativity by searching for the form that best suits the aesthetics of a product to be designed.

To implement the proposed paradigm, an agent-based architecture was chosen as it permits a modular platform to be developed and provides scalability according to users' needs. The used JADE multi-agent platform also enables its future implementation into portable devices, such as mobile phones or tablets, thanks to its compatibility with the Android operating system through the LEAP add-on for JADE [45, 46]. This portability allows designers to use the application at any time because creativity comes at no given time, nor in a specific space. So the proposed tool can be used both professionally and educationally. Despite it focusing on industrial design, its use can be extended to other fields of application.
